# Preventing corneal blindness caused by keratitis using artificial intelligence

**DOI:** 10.1038/s41467-021-24116-6

**Published:** 2021-06-18

**Authors:** Zhongwen Li, Jiewei Jiang, Kuan Chen, Qianqian Chen, Qinxiang Zheng, Xiaotian Liu, Hongfei Weng, Shanjun Wu, Wei Chen

**Affiliations:** 1 Ningbo Eye Hospital, Wenzhou Medical University, Ningbo, China; 2 School of Ophthalmology and Optometry and Eye Hospital, Wenzhou Medical University, Wenzhou, China; 3 School of Electronic Engineering, Xi’an University of Posts and Telecommunications, Xi’an, China

**Keywords:** Machine learning, Eye diseases, Disease prevention

## Abstract

Keratitis is the main cause of corneal blindness worldwide. Most vision loss caused by keratitis can be avoidable via early detection and treatment. The diagnosis of keratitis often requires skilled ophthalmologists. However, the world is short of ophthalmologists, especially in resource-limited settings, making the early diagnosis of keratitis challenging. Here, we develop a deep learning system for the automated classification of keratitis, other cornea abnormalities, and normal cornea based on 6,567 slit-lamp images. Our system exhibits remarkable performance in cornea images captured by the different types of digital slit lamp cameras and a smartphone with the super macro mode (all AUCs>0.96). The comparable sensitivity and specificity in keratitis detection are observed between the system and experienced cornea specialists. Our system has the potential to be applied to both digital slit lamp cameras and smartphones to promote the early diagnosis and treatment of keratitis, preventing the corneal blindness caused by keratitis.

## Abstract (web summary)

Keratitis is the main cause of corneal blindness worldwide, but most vision loss caused by keratitis can be avoidable via early detection and treatment, which are challenging in resource-limited settings. Here, the authors develop a deep learning system for the automated classification of keratitis and other cornea abnormalities.

## Introduction

Corneal blindness that largely results from keratitis is the fifth leading cause of global blindness, often affecting marginalized populations^1–3^. The burden of corneal blindness on people can be huge, particularly as it tends to affect an individual at a relatively younger age than other blinding reasons such as cataract and glaucoma^4^. Early detection and timely medical intervention of keratitis can deter and halt the disease progression, reaching a better prognosis, visual acuity, and even preservation of the ocular integrity^3,5–7^. Otherwise, keratitis can get worse rapidly with time, potentially leading to permanent vision loss and even corneal perforation^8,9^.

The diagnosis of keratitis often requires a skilled ophthalmologist to examine patients’ cornea through a slit-lamp microscope or slit-lamp images^10^. However, although over 200,000 ophthalmologists around the world, there is a current and expected future shortfall in the number of ophthalmologists in both developing and developed countries^11^. This widening gap between need and supply can affect the detection of keratitis in a timely manner, especially in remote and underserved regions^12^.

Recent advances in artificial intelligence (AI) and particularly deep learning have shown great promise for detecting some common diseases based on clinical images^13–15^. In ophthalmology, most studies have developed high-accuracy AI systems using fundus images for automated posterior segment disease screening, such as diabetic retinopathy, glaucoma, retinal breaks, and retinal detachment^16–22^. However, anterior segment diseases, particularly various types of keratitis, which also require prompt diagnosis and referral, are not well investigated.

Corneal blindness caused by keratitis can be completely prevented via early detection and timely treatment^8,12^. To achieve this goal, in this study, we developed a deep learning system for the automated classification of keratitis, other cornea abnormalities, and normal cornea based on slit-lamp images and externally evaluated this system in three datasets of slit-lamp images and one dataset of smartphone images. Besides, we compared the performance of this system to that of cornea specialists of different levels.

## Results

### Characteristics of the datasets

After removing 1197 images without sufficient diagnostic certainty and 594 poor-quality images, a total of 13,557 qualified images (6,055 images of keratitis, 2777 images of cornea with other abnormalities, and 4725 images of normal cornea) from 7988 individuals were used to develop and externally evaluate the deep learning system. Further information on datasets from the Ningbo Eye Hospital (NEH), Zhejiang Eye Hospital (ZEH), Jiangdong Eye Hospital (JEH), Ningbo Ophthalmic Center (NOC), and smartphone is summarized in Table 1.

**Table 1:** Characteristics of datasets.

Item		NEH dataset		ZEH dataset	JEH dataset	NOC dataset	Smartphone dataset
Total no. of images		7120		1182	2357	3386	1303
Total no. of qualified images^a^		6567		929	1987	2928	1146
No. of subjects		3568		656	1232	1849	683
Age, mean/range (years)		41.6/4–98		39.2/10–83	42.3/8–96	45.7/5–89	44.3/5–90
No. (%) of women		1689 (47.3)		302 (54.3)	533 (51.6)	799 (48.5)	344 (50.4)
Level of institution		Tertiary eye care center		Tertiary eye care center	Secondary eye care center	Secondary eye care center	Tertiary eye care center
Location of institution		Urban		Urban	Urban	Urban	Urban
Average yearly temperature (°C)		18.3		18.8	16.2	18.0	19.3
Camera model		Canton Optics LS-7 (China)		Sanyo VPC-MZ3GX (Japan)	Kanghua SLM-3 (China)	Nikon DSC D5200 (Japan)	Huawei P30 (China)
	**Training set**	**Validation set**	**Test set**				
Keratitis^b^	2185/4526 (48.3)	511/1055 (48.4)	483/986 (49.0)	378/929 (40.7)	1186/1987 (59.7)	843/2928 (28.8)	469/1146 (40.9)
Cornea with other abnormalities^b^	585/4526 (12.9)	136/1055 (12.9)	130/986 (13.2)	237/929 (25.5)	236/1987 (11.9)	969/2928 (33.1)	484/1146 (42.2)
Normal cornea^b^	1756/4526 (38.8)	408/1055 (38.7)	373/986 (37.8)	314/929 (33.8)	565/1987 (28.4)	1116/2928 (38.1)	193/1146 (16.9)

*NEH* Ningbo Eye Hospital, *ZEH* Zhejiang Eye Hospital, *JEH* Jiangdong Eye Hospital, *NOC* Ningbo Ophthalmic Center.

^a^Qualified images indicate the images with sufficient diagnostic certainty and good quality.

^b^Data are no. of images/total no. (%) unless otherwise indicated.

### Performance of different deep learning algorithms in the internal test dataset

**Fig. 1:**
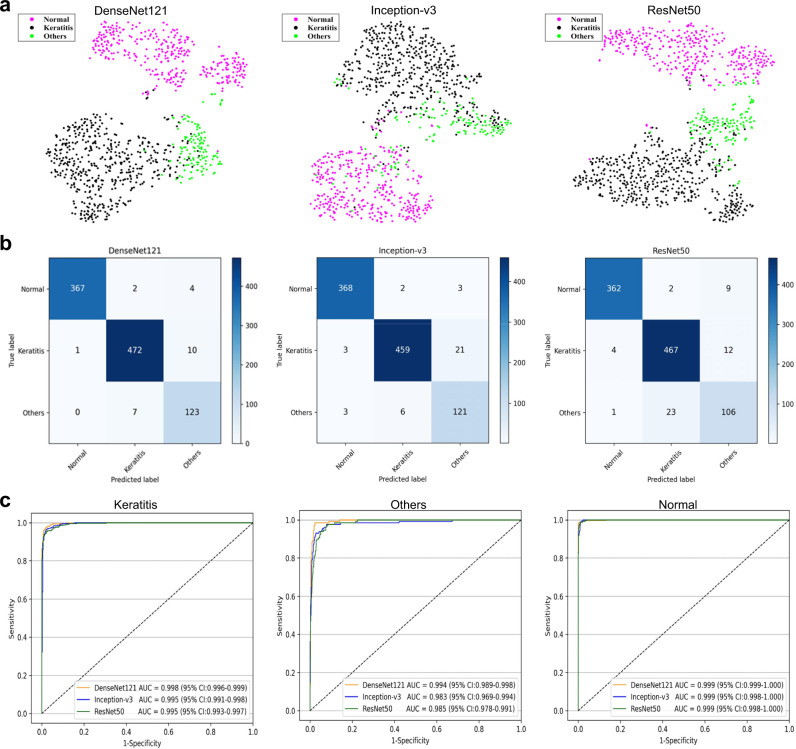
Performance of deep learning algorithms in the internal test dataset from Ningbo Eye Hospital. **a** Visualization by t-distributed stochastic neighbor embedding (t-SNE) of the separability for the features learned by deep learning algorithms. Different colored point clouds represent the different categories. **b** Confusion matrices describing the accuracies of three deep learning algorithms. **c** Receiver operating characteristic curves indicating the performance of each algorithm for detecting keratitis, cornea with other abnormalities, and normal cornea. “Normal” indicates normal cornea. “Others” indicates cornea with other abnormalities.

Three classic deep learning algorithms, DenseNet121, Inception-v3, and ResNet50, were used in this study to train models for the classification of keratitis, cornea with other abnormalities, and normal cornea. The t-distributed stochastic neighbor embedding (t-SNE) technique indicated that the features of each category learned by the DenseNet121 algorithm were more separable than those of the Inception-v3 and ResNet50 (Fig. 1a). The performance of these three algorithms in the internal test dataset is described in Fig. 1b and c, which illustrate that the best algorithm is the DenseNet121. Further information including accuracies, sensitivities, and specificities of these algorithms is shown in Table 2. The best algorithm achieved an area under the curve (AUC) of 0.998 (95% confidence interval [CI], 0.996–0.999), a sensitivity of 97.7% (95% CI, 96.4–99.1), and a specificity of 98.2% (95% CI, 97.1–99.4) in keratitis detection. The best algorithm discriminated cornea with other abnormalities from keratitis and normal cornea with an AUC of 0.994 (95% CI, 0.989–0.998), a sensitivity of 94.6% (95% CI, 90.7–98.5), and a specificity of 98.4% (95% CI, 97.5–99.2). The best algorithm discriminated normal cornea from abnormal cornea (including keratitis and other cornea abnormalities) with an AUC of 0.999 (95% CI, 0.999–1.000), a sensitivity of 98.4% (95% CI, 97.1–99.7), and a specificity of 99.8% (95% CI, 99.5–100). Compared to the reference standard of the internal test dataset, the unweighted Cohen’s kappa coefficient of the best algorithm DenseNet121 was 0.960 (95% CI: 0.944–0.976).

**Table 2:** Performance of three deep learning algorithms in the internal test dataset.

One-vs.-rest classification	NEH internal test dataset		
	Sensitivity (95% CI)	Specificity (95% CI)	Accuracy (95% CI)
Keratitis vs. others + normal			
DenseNet121	97.7% (96.4–99.1)	98.2% (97.1–99.4)	98.0% (97.1–98.9)
Inception-v3	95.0% (93.1–97.0)	98.4% (97.3–99.5)	96.8% (95.6–97.9)
ResNet50	96.7% (95.1–98.3)	95.0% (93.1–96.9)	95.8% (94.6–97.1)
Others vs. keratitis + normal			
DenseNet121	94.6% (90.7–98.5)	98.4% (97.5–99.2)	97.9% (97.0–98.8)
Inception-v3	93.1% (88.7–97.4)	97.2% (96.1–98.3)	96.7% (95.5–97.8)
ResNet50	81.5% (74.9–88.2)	97.5% (96.5–98.6)	95.4% (94.1–96.7)
Normal vs. keratitis + others			
DenseNet121	98.4% (97.1–99.7)	99.8% (99.5–100)	99.3% (98.8–99.8)
Inception-v3	98.7% (97.5–99.8)	99.0% (98.2–99.8)	98.9% (98.2–99.5)
ResNet50	97.1% (95.3–98.8)	99.2% (98.5–99.9)	98.4% (97.6–99.2)

“Normal” indicates normal cornea. “Others” indicates cornea with other abnormalities. NEH, Ningbo Eye Hospital. CI, confidence interval.

*NEH* Ningbo Eye Hospital, *CI* confidence interval.

### Performance of different deep learning algorithms in the external test datasets

**Fig. 2:**
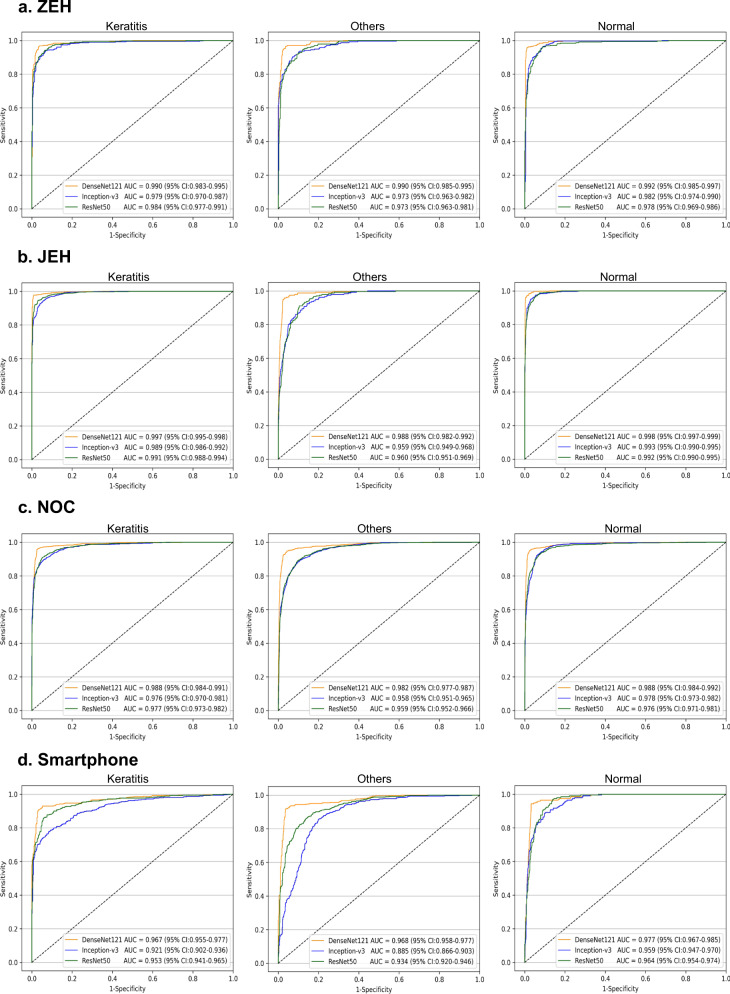
Receiver operating characteristic curves of three deep learning algorithms in the external test datasets. **a** Zhejiang Eye Hospital (ZEH) dataset. **b** Jiangdong Eye Hospital (JEH) dataset. **c** Ningbo Ophthalmic Center (NOC) dataset. **d** “Smartphone” indicates the smartphone dataset. “Normal” indicates normal cornea. “Others” indicates cornea with other abnormalities.

In the external test datasets, the t-SNE technique also showed that the features of each category learned by the DenseNet121 algorithm were more separable than those of Inception-v3 and ResNet50 (Supplementary Fig. 1). Correspondingly, the receiver operating characteristic (ROC) curves (Fig. 2) and the confusion matrices (Supplementary Fig. 2) of these algorithms in the external datasets indicated that the DenseNet121 algorithm has the best performance in the classification of keratitis, cornea with other abnormalities, and normal cornea.

In the ZEH dataset, the best algorithm achieved AUCs of 0.990 (95% CI, 0.983–0.995), 0.990 (95% CI, 0.985–0.995), and 0.992 (95% CI, 0.985–0.997) for the classification of keratitis, cornea with other abnormalities, and normal cornea, respectively. In the JEH dataset, the best algorithm achieved AUCs of 0.997 (95% CI, 0.995–0.998), 0.988 (95% CI, 0.982–0.992), and 0.998 (95% CI, 0.997–0.999) for the classification of keratitis, cornea with other abnormalities, and normal cornea, respectively. In the NOC dataset, the best algorithm achieved AUCs of 0.988 (95% CI, 0.984–0.991), 0.982 (95% CI, 0.977–0.987), and 0.988 (95% CI, 0.984–0.992) for the classification of keratitis, cornea with other abnormalities, and normal cornea, respectively.

In the smartphone dataset, the DenseNet121 algorithm still showed the best performance in detecting keratitis, cornea with other abnormalities, and normal cornea. The best algorithm achieved an AUC of 0.967 (95% CI, 0.955–0.977), a sensitivity of 91.9% (95% CI, 89.4–94.4), and a specificity of 96.9% (95% CI, 95.6–98.2) in keratitis detection. The best algorithm discriminated cornea with other abnormalities from keratitis and normal cornea with an AUC of 0.968 (95% CI, 0.958–0.977), a sensitivity of 93.4% (95% CI, 91.2–95.6), and a specificity of 95.6% (95% CI, 94.1–97.2). The best algorithm discriminated normal cornea from abnormal cornea (including keratitis and cornea with other abnormalities) with an AUC of 0.977 (95% CI, 0.967–0.985), a sensitivity of 94.8% (95% CI, 91.7–97.9), and a specificity of 96.9% (95% CI, 95.7–98.0).

The details on the performance of each algorithm (DenseNet121, Inception-v3, and ResNet50) in the external test datasets are shown in Table 3. Compared to the reference standard of the ZEH dataset, JEH dataset, NOC dataset, and smartphone dataset, the unweighted Cohen’s kappa coefficients of the best algorithm DenseNet121 were 0.933 (95% CI, 0.913–0.953), 0.947 (95% CI, 0.934–0.961), 0.926 (95% CI, 0.915–0.938), and 0.889 (95% CI, 0.866–0.913), respectively.

**Table 3:** Performance of three deep learning algorithms in the external test datasets.

One-vs.-rest classification	ZEH external test dataset			JEH external test dataset			NOC external test dataset			Smartphone-based external test dataset		
	Sensitivity (95% CI)	Specificity (95% CI)	Accuracy (95% CI)	Sensitivity (95% CI)	Specificity (95% CI)	Accuracy (95% CI)	Sensitivity (95% CI)	Specificity (95% CI)	Accuracy (95% CI)	Sensitivity (95% CI)	Specificity (95% CI)	Accuracy (95% CI)
Keratitis vs. others + normal												
DenseNet121	96.0% (94.1–98.0)	97.1% (95.7–98.5)	96.7% (95.5–97.8)	97.7% (96.9–98.6)	98.9% (98.1–99.6)	98.2% (97.6–98.8)	96.8% (95.6–98.0)	97.0% (96.3–97.8)	97.0% (96.3–97.6)	91.9% (89.4–94.4)	96.9% (95.6–98.2)	94.9% (93.6–96.1)
Inception-v3	88.9% (85.7–92.1)	96.7% (95.2–98.2)	93.5% (92.0–95.1)	92.5% (91.0–94.0)	95.0% (93.5–96.5)	93.5% (92.4–94.6)	87.1% (84.8–89.3)	96.0% (95.2–96.9)	93.4% (92.5–94.3)	70.6% (66.5–74.7)	96.5% (95.1–97.8)	85.9% (83.8–87.9)
ResNet50	90.7% (87.8–93.7)	95.6% (93.9–97.3)	93.6% (92.1–95.2)	96.3% (95.2–97.4)	93.9% (92.2–95.5)	95.3% (94.4–96.2)	92.2% (90.4–94.0)	91.9% (90.8–93.1)	92.0% (91.0–93.0)	84.6% (81.4–87.9)	94.5% (92.8–96.2)	90.5% (88.8–92.2)
Others vs. keratitis + normal												
DenseNet121	94.5% (91.6–97.4)	97.0% (95.7–98.2)	96.3% (95.1–97.5)	96.2% (93.7–98.6)	97.6% (96.9–98.3)	97.4% (96.7–98.1)	93.8% (92.3–95.3)	97.5% (96.9–98.2)	96.3% (95.6–97.0)	93.4% (91.2–95.6)	95.6% (94.1–97.2)	94.7% (93.4–96.0)
Inception-v3	91.6% (88.0–95.1)	91.0% (88.9–93.2)	91.2% (89.3–93.0)	84.7% (80.2–89.3)	91.8% (90.5–93.1)	90.9% (89.7–92.2)	87.2% (85.1–89.3)	89.7% (88.3–91.0)	88.9% (87.7–90.0)	84.7% (81.5–87.9)	80.5% (77.5–83.5)	82.3% (80.1–84.5)
ResNet50	92.8% (89.5–96.1)	90.0% (87.8–92.3)	90.7% (88.9–92.6)	82.6% (77.8–87.5)	92.9% (91.7–94.1)	91.7% (90.5–92.9)	82.9% (80.5–85.2)	93.4% (92.3–94.5)	89.9% (88.8–91.0)	87.8% (84.9–90.7)	84.9% (82.2–87.6)	86.1% (84.1–88.1)
Normal vs. keratitis + others												
DenseNet121	95.9% (93.7–98.1)	99.3% (98.7–100)	98.2% (97.3–99.0)	96.1% (94.5–97.7)	99.5% (99.1–99.9)	98.5% (98.0–99.1)	95.0% (93.7–96.3)	98.2% (97.6–98.8)	97.0% (96.3–97.6)	94.8% (91.7–97.9)	96.9% (95.7–98.0)	96.5% (95.4–97.6)
Inception-v3	87.3% (83.6–90.9)	96.4% (95.0–97.9)	93.3% (91.7–94.9)	87.1% (84.3–89.8)	99.0% (98.5–99.5)	95.6% (94.7–96.5)	87.3% (85.3–89.2)	95.0% (94.0–96.0)	92.1% (91.1–93.1)	86.0% (81.1–90.9)	91.0% (89.2–92.8)	90.1% (88.4–91.9)
ResNet50	81.8% (77.6–86.1)	97.4% (96.1–98.7)	92.1% (90.4–93.9)	81.9% (78.8–85.1)	99.0% (98.5–99.5)	94.2% (93.1–95.2)	87.0% (85.0–89.0)	95.6% (94.6–96.5)	92.3% (91.4–93.3)	74.1% (67.9–80.3)	95.4% (94.1–96.7)	91.8% (90.2–93.4)

“Normal” indicates normal cornea. “Others” indicates cornea with other abnormalities.

*CI* confidence interval, *NEH* Ningbo Eye Hospital, *ZEH* Zhejiang Eye Hospital, *JEH* Jiangdong Eye Hospital, *NOC* Ningbo Ophthalmic Center.

The performance of the best algorithm DenseNet121 in the external test datasets with and without poor-quality images is described in Supplementary Fig. 4. The AUCs of the best algorithm in the datasets with poor-quality images were slightly lower than the datasets without poor-quality images. Besides, a total of 168 images were assigned to the category of mild keratitis. The best algorithm DenseNet121 achieved an accuracy of 92.3% (155/168) in identifying mild keratitis.

### Classification errors

**Fig. 3:**
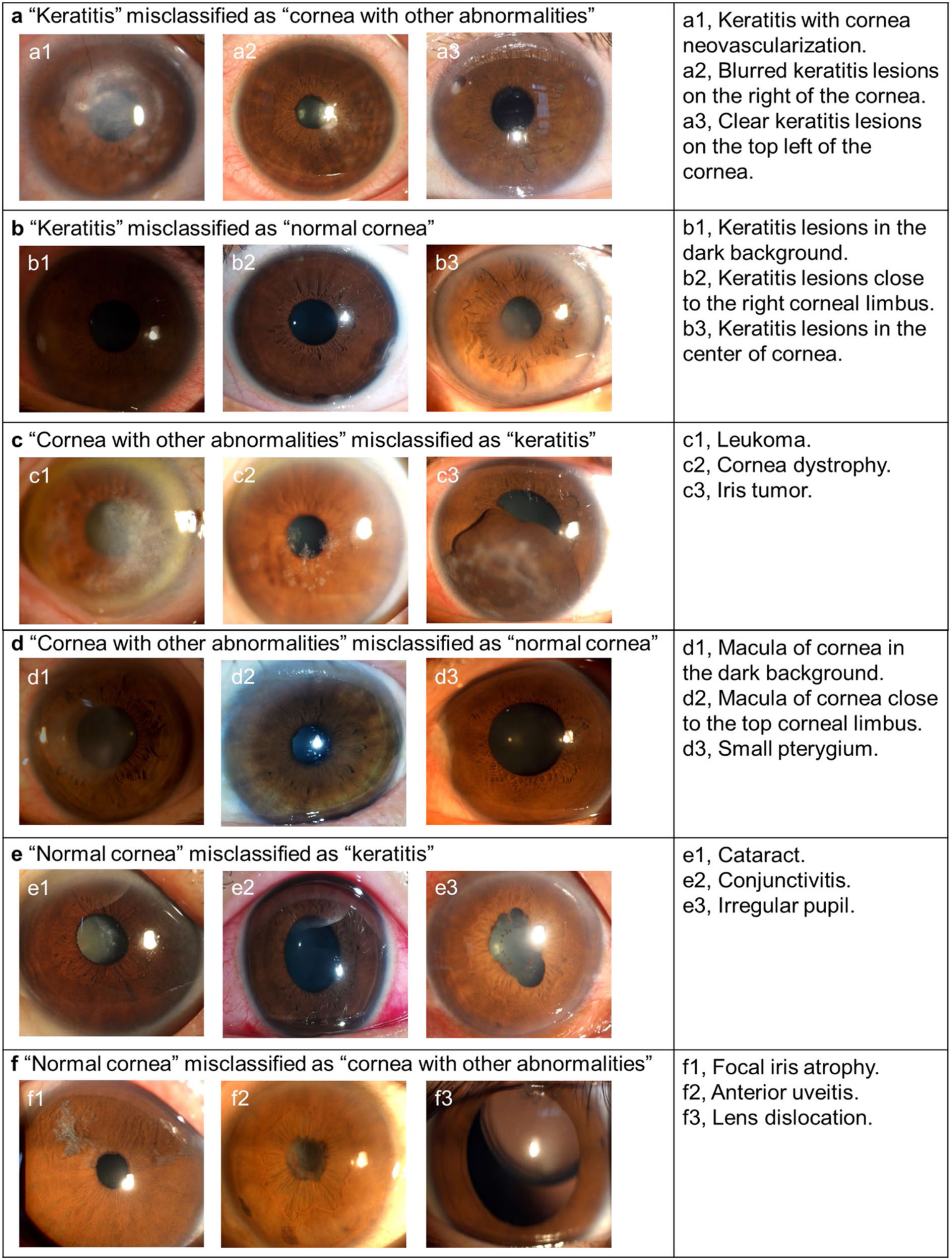
Typical examples of misclassified images by the deep learning system. **a** Images of “keratitis” incorrectly classified as “cornea with other abnormalities”. **b** Images of “keratitis” incorrectly classified as “normal cornea”. **c** Images of “cornea with other abnormalities” incorrectly classified as “keratitis”. **d** Images of “cornea with other abnormalities” incorrectly classified as “normal cornea”. **e** Images of “normal cornea” incorrectly classified as “keratitis”. **f** Images of “normal cornea” incorrectly classified as “cornea with other abnormalities”.

In both internal and external test datasets, a total of 346 images (4.3% of the 7976 images) had discordant findings between the deep learning system and the reference standard. In the category of keratitis (3359), 87 images (2.6%) were misclassified by the system as cornea with other abnormalities, and 31 images (0.9%) were misclassified as the normal cornea. For the keratitis incorrectly classified as cornea with other abnormalities, 56.3% (49/87) images showed keratitis with cornea neovascularization. These cases often have similar features of the pterygium, and it might be a possible contributor for this misclassification. For the keratitis misclassified as the normal cornea, 54.8% (17/31) images were underexposed, affecting the clarity of the lesions. In the category of cornea with other abnormalities (2056 images), 77 images (3.8%) were misclassified by the system as keratitis and 44 images (2.1%) were misclassified as normal cornea. For the cornea with other abnormalities misclassified as keratitis, 76.6% (59/77) images showed leukoma and macula which is similar to the features of the keratitis at the reparative phase. For the cornea with other abnormalities misclassified as the normal cornea, the most common reason was the small lesion of keratitis close to the corneal limbus, which was shown in 50% (22/44) images. In the category of normal cornea (2,561 images), 40 images (1.6%) were misclassified by the system as keratitis and 67 images (2.6%) were misclassified as cornea with other abnormalities. For the normal cornea incorrectly classified as keratitis and cornea with other abnormalities, over half of images (57.9%, 62/107) had cataracts. The appearance of cataract in a two-dimensional image often resembles that of some keratitis and leukoma located at the center of the cornea. The details regarding classification errors by the deep learning system are described in Supplementary Fig. 3. Typical examples of misclassified images are shown in Fig. 3.

The relationship between the misclassification rates and predicted probability values of the best algorithm DenseNet121 was shown in Supplementary Fig. 5, which indicated that the misclassification rates of each category and total misclassification rates both increased with the decline of the predicted probability values. When the predicted probabilities are greater than 0.866, the misclassification rates for all categories are less than 3%. When the probabilities are less than 0.598, the misclassification rates of the normal cornea are about 12% and the misclassification rates of the other two categories are greater than 20%. As our model is a three-category classification model, the lowest predicted probability value of the model’s output is greater than 0.33.

### Heatmaps

**Fig. 4:**
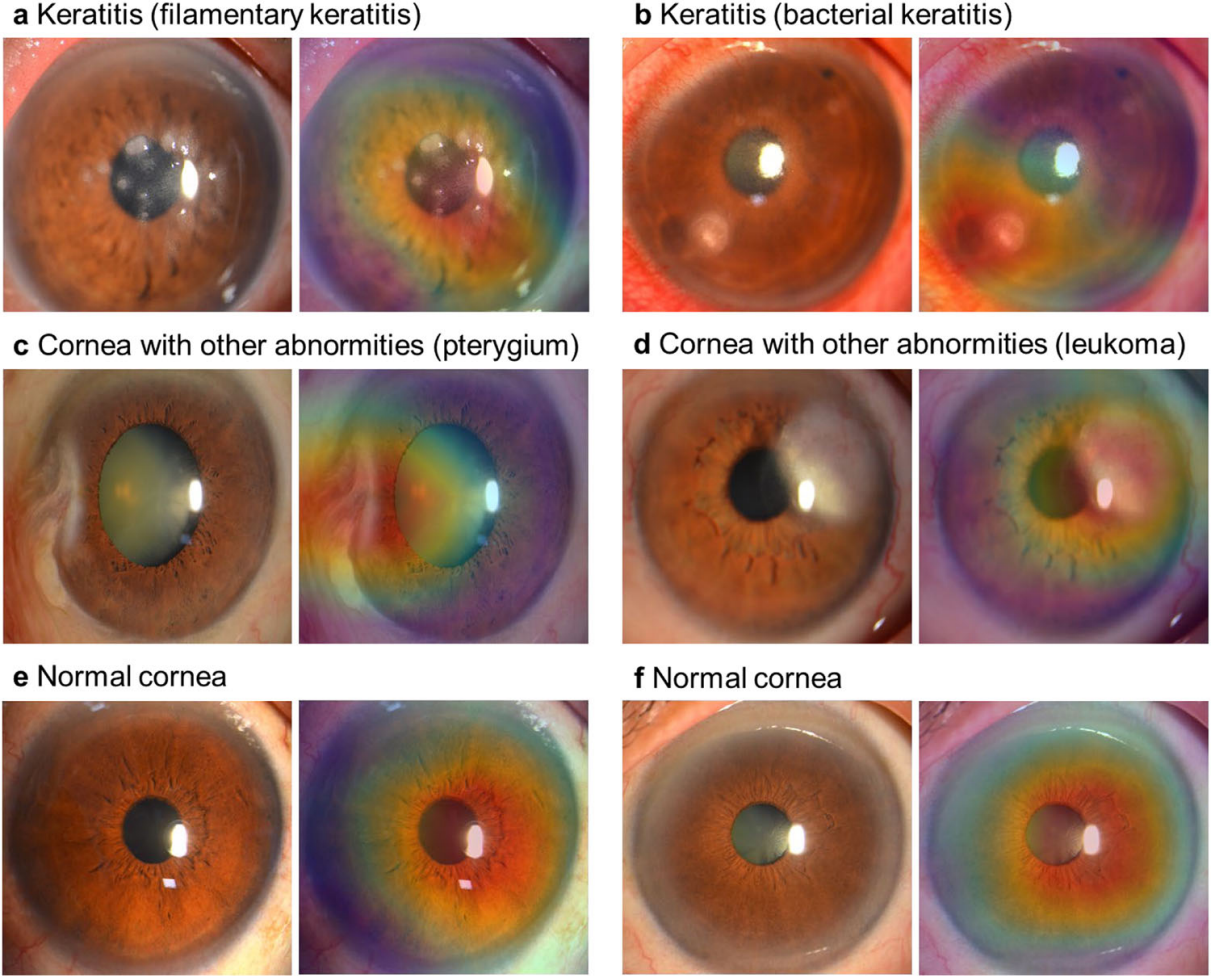
Heatmaps demonstrating typical findings, shown in pairs of original images (left) and corresponding heatmaps (right) for each category. **a** Filamentary keratitis. **b** Bacterial keratitis. **c** Pterygium. **d** Leukoma. **e**, **f** Normal cornea.

To visualize the regions contributing most to the system, we generated a heatmap that superimposed a visualization layer at the end of the convolutional neural network (CNN). For abnormal cornea findings (including keratitis and cornea with other abnormalities), heatmaps effectively highlighted the lesion regions. For normal cornea, heatmaps displayed highlighted visualization on the region of the cornea. Typical examples of the heatmaps for keratitis, cornea with other abnormalities, and normal cornea are presented in Fig. 4.

### Comparison of the deep learning system against corneal specialists

In the ZEH dataset, for the classification of keratitis, cornea with other abnormalities, and normal cornea, the cornea specialist with 3 years of experience achieved accuracies of 96.2% (95.0–97.5), 95.2% (93.8–96.5), and 98.3% (97.4–99.1), respectively, and the senior cornea specialist with 6 years of experience achieved accuracies of 97.3% (96.3–98.3), 96.6% (95.4–97.7), and 98.6% (97.8–99.4), respectively, while the deep learning system achieved accuracies of 96.7% (95.5–97.8), 96.3% (95.1–97.5), and 98.2% (97.3–99.0), respectively. The performance of our system is comparable to that of the cornea specialists (På 0.05) (Supplementary Table 1).

## Discussion

In this study, our purpose was to evaluate the performance of a deep learning system to detect keratitis from slit-lamp images taken at multiple clinical institutions using different commercially available digital slit-lamp cameras. Our main finding was that the system based on deep learning neural networks could discriminate among keratitis, cornea with other abnormalities, and normal cornea and the DenseNet121 algorithm had the best performance. In our three external test datasets consisting of slit-lamp images, the sensitivity for detecting keratitis was 96.0–97.7% and the specificity was 96.7–98.2%, which demonstrated the broad generalizability of our system. In addition, the unweighted Cohen’s Kappa coefficients showed a high agreement between the outcomes of the deep learning system and the reference standard (all over 0.88), further substantiating the effectiveness of our system. Moreover, our system exhibited comparable performance to that of cornea specialists in the classification of keratitis, cornea with other abnormalities, and normal cornea.

In less developed communities, corneal blindness is associated with older age, the lack of education, and being occupied in farming and outdoor jobs^12,23^. People there show little knowledge and awareness about keratitis and few of them choose to go to the hospital when they have symptoms of keratitis (e.g., eye pain and red eyes)^4,24^. Patients usually present for treatment only after the corneal ulcer is well established and visual acuity is severely compromised^4,25^. In addition, less eye care service in these regions (low ratio of eye doctors per 10,000 inhabitants) is another important reason that prevents patients with keratitis from visiting eye doctors in a timely manner^11,12,23^. Therefore, the corneal blindness rate in these underserved communities is often high. As an automated screening tool, the system developed in this study can be applied in the aforementioned communities for identifying the keratitis at an early stage and providing a timely referral for the positive cases, which has the potential to prevent corneal blindness caused by keratitis.

For the cornea images that were captured by a smartphone with super macro mode, our system still performed well in detecting keratitis, cornea with other abnormalities, and normal cornea (all accuracies over 94%). This result indicates that we have the potential to apply our system to smartphones, which would be a cost-effective and convenient procedure for the early detection of keratitis, making it especially suitable for the high-risk people, such as farmers who live in resource-limited settings and the people who often wear contact lens^4,26,27^.

**Fig. 5:**
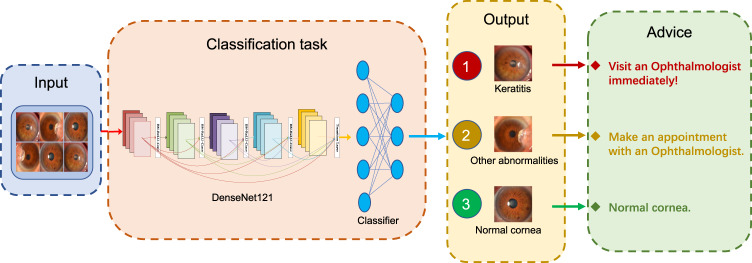
Workflow of the deep learning system in clinics for detecting abnormal cornea findings. Patients with keratitis detected by the system are advised to visit ophthalmologists immediately. Patients with other cornea abnormalities detected by the system are advised to make an appointment with ophthalmologists.

Keratitis, especially microbial keratitis, is an ophthalmic emergency that requires immediate attention because it can progress rapidly, even results in blindness^9,28,29^. The faster patients receive treatment, the less likely they are to have serious and long-lasting complications^29^. Therefore, our system is set to inform patients to visit ophthalmologists immediately if their cornea images are identified to have keratitis. For the image of other cornea abnormalities, our system will advise the corresponding patients to make an appointment with ophthalmologists to clarify whether they need further examination and treatment. The workflow of our system is described in Fig. 5.

Recently, several reports of automated approaches for keratitis detection have been published. Gu et al.^30^ established a deep learning system that could detect keratitis with an AUC of 0.93 in 510 slit-lamp images. Kuo et al.^31^ used a deep learning approach for discerning fungal keratitis based on 288 corneal photographs, reporting an AUC of 0.65. Loo et al.^32^ proposed a deep learning-based algorithm to identify and segment ocular structures and microbial keratitis biomarkers on slit-lamp images and the Dice similarity coefficients of the algorithm for all regions of interests ranged from 0.62 to 0.85 on 133 eyes. Lv et al.^33^ established an intelligent system based on deep learning for automatically diagnosing fungal keratitis using 2088 in vivo confocal microscopy images and their system reached an accuracy of 96.2% in detecting fungal hyphae. When compared to the previous studies, our study had a number of important features. First, for the screening purpose, we established a robust deep learning system that could automatically detect keratitis and other cornea abnormalities from both slit-lamp images (all AUCs over 0.98) and smartphone images (all AUCs over 0.96). Second, to enhance the performance of our system, the datasets that we utilized to train and verify the system were substantially larger (13,557 images from 7988 individuals) than those of previous studies. Finally, our datasets were acquired at four clinical centers with different types of cameras and thereby were more representative of data in the real world.

To make the output of our deep learning system interpretable, heatmaps were generated to visualize where the system attended to for the final decisions. In the heatmaps of keratitis and other cornea abnormalities, the regions of cornea lesions were highlighted. In the heatmaps of normal cornea, the highlighted region was colocalized with almost the entire cornea region. This interpretability feature of our system could further promote its application in real-world settings as ophthalmologists can understand how the final output is made by the system.

Although our system had robust performance, misclassification still existed. The relationship between the misclassification rate and predicted probability of the system was analyzed and the results indicated that the lower the predicted probability is, the higher the misclassification rate is. Therefore, the image with a low predicted probability value needs the attention of a cornea specialist. An ideal AI system should minimize the number of false results. We expect more studies to investigate how this happened and to find strategies to minimize errors.

Our study has several limitations. First, two-dimensional images rather than three-dimensional images were used to train the deep learning system, thus making a few misclassifications due to the image lacking stereoscopic quality. For example, in two-dimensional images, some normal cornea images with cataract were misclassified as keratitis probably because the white cloudy area of keratitis in some cases appeared in the center of the cornea, which was similar to the appearance of the cataract with the normal cornea. Second, our system cannot make a specific diagnosis based on a slit-lamp image or a smartphone image. Notably, for the screening purpose, it is more reasonable and reliable to detect keratitis instead of specifying the type of keratitis only based on an image without considering other clinical information (e.g., age, predisposing factors, and medical history) and examination^7^. In addition, the case with keratitis infected by multiple microbes (e.g., bacteria, fungus, and ameba) is not uncommon in clinics, which is difficult to be diagnosed merely through a cornea image. Third, due to a limited number of poor-quality images in the development dataset, this study did not develop a deep learning-based image quality control system to detect and filter out poor-quality images, which may negatively affect the subsequent AI diagnostic systems. Our research group will keep collecting more poor-quality images and further develop an independent image quality control system in the near future. Fourth, as eyes with keratitis in a subclinical stage often don’t show clinical manifestations (signs and/or symptoms), patients with subclinical keratitis rarely visit eye doctors, making the collection of subclinical keratitis images difficult. Therefore, this study did not evaluate the performance of the system in identifying subclinical keratitis due to the lack of subclinical keratitis images. Instead, we evaluated the performance of our system in detecting mild keratitis, which could usually be effectively treated without loss of vision.

In conclusion, we developed a deep learning system that could accurately detect keratitis, cornea with other abnormalities, and normal cornea from both slit-lamp and smartphone images. As a preliminary screening tool, our system has the high potential to be applied to digital slit-lamp cameras and smartphones with super macro mode for the early diagnosis of keratitis in resource-limited settings, reducing the incidence of corneal blindness.

## Methods

### Image datasets

In this study, a total of 7120 slit-lamp images (2584 × 2000 pixels in JPG format) that were consecutively collected from 3568 individuals at NEH between January 2017 and March 2020 were employed to develop a deep learning system. The NEH dataset included individuals who presented for ocular surface disease examination, ophthalmology consultations, and routine ophthalmic health evaluations. The images were captured under diffused illumination using a digital slit-lamp camera.

Three additional datasets encompassing 6925 slit-lamp images drawn from three other institutions were utilized to externally test the system. One was collected from the outpatient clinics, inpatient department, and dry eye center at ZEH, consisting of 1182 images (2592 × 1728 pixels in JPG format) from 656 individuals; one was collected from outpatient clinics and health screening center at JEH, consisting of 2357 images (5784 × 3456 pixels in JPG format) from 1232 individuals; and the remaining one was collected from the outpatient clinics and inpatient department at NOC, consisting of 3386 images (1740 × 1536 pixels in PNG format) from 1849 individuals.

**Fig. 6:**
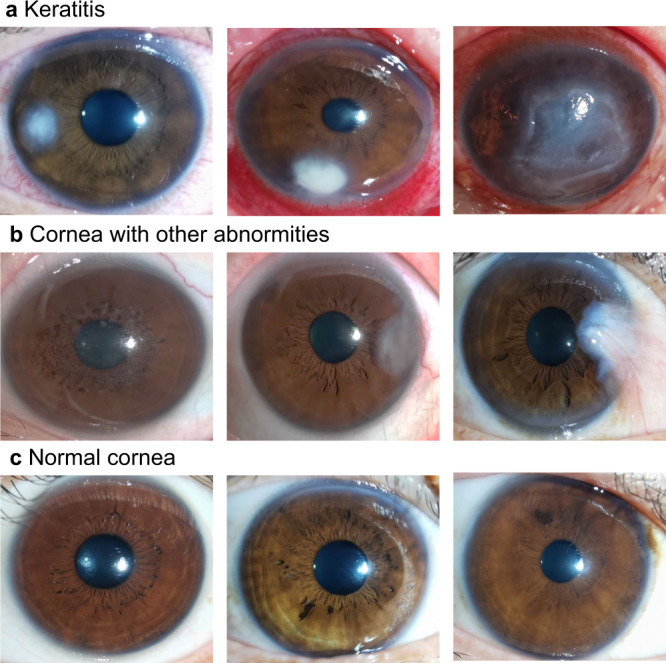
Typical examples of the smartphone-based cornea images. **a** Keratitis. **b** Cornea with other abnormalities: the left image shows cornea dystrophy, the middle image shows leukoma, and the right image shows pterygium. **c** Normal cornea.

Besides, 1303 smartphone-based cornea images (3085 × 2314 pixels in JPG format) from 683 individuals were collected as one of the external test datasets. This smartphone dataset was derived from Wenzhou Eye Study which aimed to detect ocular surface diseases using smartphones. These images were captured using the super macro mode of HUAWEI P30 through the following standard steps: (1) Open super macro mode and camera flash; (2) Put the rear camera 2–3 cm in front of the cornea; (3) Ask individuals to look straight ahead and open their both eyes as wide as possible; (4) Take an image when the focus is on the cornea. Typical examples of the smartphone-based cornea images were shown in Fig. 6.

All deidentified, unaltered images (size, 1–6 megabytes per image) were transferred to research investigators for inclusion in the study. The study was approved by the Institution Review Board of NEH (identifier, 2020-qtky-017) and adhered to the principles of the Declaration of Helsinki. Informed consent was exempted, due to the retrospective nature of the data acquisition and the use of deidentified images.

### Reference standard and image classification

A specific diagnosis provided by cornea specialists for each slit-lamp image was based on the clinical manifestations, corneal examination (e.g., fluorescein staining of the cornea, corneal confocal microscopy, and specular microscopy), laboratory methods (e.g., corneal scraping smear examination, the culture of corneal samples, PCR, and genetic analyses), and follow-up visits. The diagnosis based on these medical records was considered as the reference standard of this research. Our ophthalmologists (ZL and KC) independently reviewed all data in detail before any analyses and validated that each image was correctly matched to a specific individual. Images without sufficient evidence to determine a diagnosis were excluded from the study.

All images with sufficient diagnostic certainty were screened for quality control. Poor-quality and unreadable images were excluded. The qualified images were classified by the study steering committee into three categories, consistent with the reference diagnosis: keratitis caused by infectious and/or noninfectious factors, cornea with other abnormalities, and normal cornea. Infectious keratitis included bacterial keratitis, fungal keratitis, viral keratitis, parasitic keratitis, etc. Noninfectious keratitis included ultraviolet keratitis, inflammation from eye injuries or chemicals, autoimmune keratitis, etc. The cornea with other abnormalities includes corneal dystrophies, corneal degeneration, corneal tumors, pterygium, etc.

### Image preprocessing

During the image preprocessing phase, standardization was performed to downsize the image to 224 × 224 pixels and normalize the pixel values from 0 to 1. Afterward, data augmentation techniques were applied to increase the diversity of the dataset and thus alleviate the overfitting problem in the deep learning process. The new samples were generated through the simple transformations of original images, which was consistent with “real-world” acquisition conditions. Random cropping, horizontal and vertical flipping, and rotations were applied to the images of the training dataset to increase the sample size to six times the original size (from 4526 to 27,156).

### Development and evaluation of the deep learning system

The slit-lamp images drawn from the NEH dataset were randomly divided (7:1.5:1.5) into training, validation, and test datasets. Images from the same individual were assigned to only one same set for preventing leakage and biased assessment of performance. The training and validation datasets were used to develop the system and the test dataset was used to evaluate the performance of the system.

For obtaining the best deep learning model to classify cornea into one of the three categories: keratitis, cornea with other abnormalities, and normal cornea, three state-of-the-art CNN architectures (DenseNet121, Inception-v3, and ResNet50) were investigated in this study. Weights pre-trained for ImageNet classification were employed to initialize the CNN architectures^34^.

Deep learning models were trained using PyTorch (version 1.6.0) as a backend. The adaptive moment estimation (ADAM) optimizer with a 0.001 initial learning rate, β1 of 0.9, β2 of 0.999, and weight decay of 1e-4 was used. Each model was trained for 80 epochs. During the training process, validation loss was assessed on the validation dataset after each epoch and used as a reference for model selection. Each time the validation loss decreased, a checkpoint saved the model state and corresponding weight matrix. The model state with the lowest validation loss was saved as the final state of the model for use on the test dataset.

**Fig. 7:**
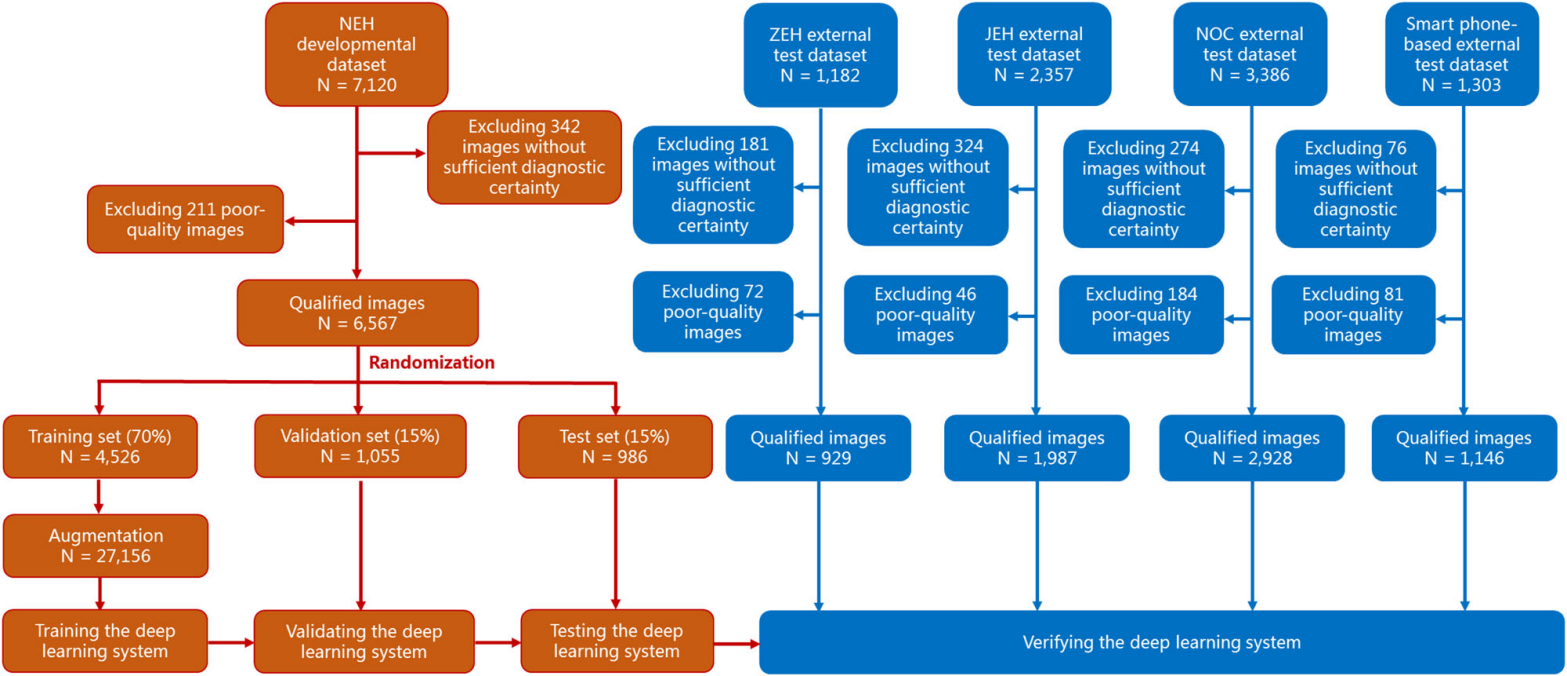
Flow chart for the development and evaluation of the deep learning system. NEH Ningbo Eye Hospital, ZEH Zhejiang Eye Hospital, JEH Jiangdong Eye Hospital, NOC Ningbo Ophthalmic Center.

The diagnostic performance of the three-category classification model was then evaluated on four independent external test datasets. The process of the development and evaluation of the deep learning system is illustrated in Fig. 7. The t-SNE technique was used to display the embedding features of each category learned by the deep learning model in a two-dimensional space^35^. In addition, the performance of the model on the external test datasets that included poor-quality images was also assessed.

As detecting keratitis at an early stage (mild keratitis) when clinical features were not obvious was critical for improving vision prognosis, all the mild keratitis images were screened out manually from external test datasets in terms of the criteria used to grade the severity of keratitis cases (mild: lesion outside central 4 mm, <2 mm in diameter)^36,37^ and the performance of the model in identifying mild keratitis was evaluated.

### Visualization heatmap

Gradient-weighted Class Activation Mapping (Grad-CAM) technique was employed to produce “visual explanations” for decisions from the system. This technique uses the gradients of any target concept, flowing into the last convolutional layer to produce a localization map highlighting important areas in the image for predicting the concept^38^. Redder regions represent more significant features of the system’s classification. Using this approach, the heatmap was generated to illustrate the rationale of the deep learning system on the discrimination among keratitis, cornea with other abnormalities, and normal cornea.

### Characteristics of misclassification by the deep learning system

In a post-hoc analysis, a senior corneal specialist reviewed all misclassified images made by the deep learning system. To interpret these discrepancies, the possible reasons for the misclassification were analyzed and documented based on the observed characteristics from the images. Besides, the relationship between the misclassification rate and predicted probability of the system was investigated.

### Deep learning versus cornea specialists

To assess our deep learning system in the context of keratitis detection, we recruited two cornea specialists who had 3 and 6 years of clinical experience. The ZEH dataset was employed to compare the performance of the best system (DenseNet121) to that of corneal specialists with the reference standard. They independently classified each image into one of the following three categories: keratitis, cornea with other abnormalities, and normal cornea. Notably, to reflect the level of the cornea specialists in normal clinical practices, they were not told that they competed against the system to avoid bias from the competition.

### Statistical analysis

The performance of the deep learning model for the classification of keratitis, cornea with other abnormalities, and normal cornea was evaluated by utilizing the one-versus-rest strategy and calculating the sensitivity, specificity, accuracy, and AUC. Statistical analyses were conducted using Python 3.7.8 (Wilmington, Delaware, USA). The 95% CIs for sensitivity, specificity, and accuracy were calculated with the Wilson Score approach using a Statsmodels package (version 0.11.1), and for AUC, using Empirical Bootstrap with 1000 replicates. We plotted the ROC curves to show the ability of the system. The ROC curve was created by plotting the ratio of true positive cases (sensitivity) against the ratio of false-positive cases (1-specificity) using the packages of Scikit-learn (version 0.23.2) and Matplotlib (version 3.3.1). A larger area under the ROC curve indicated better performance. Unweighted Cohen’s kappa coefficients were calculated to compare the results of the system to the reference standard. The differences in the sensitivities, specificities, and accuracies between the system and corneal specialists were analyzed using the McNemar test. All statistical tests were two-sided with a significance level of 0.05.

### Reporting summary

Further information on research design is available in the Nature Research Reporting Summary linked to this article.

## Supplementary information

Supplementary Information

Reporting Summary

## Data Availability

The data generated and/or analyzed during the current study are available upon reasonable request from the corresponding author. Correspondence and requests for data materials should be addressed to WC (chenwei@eye.ac.cn). The data can be accessed only for research purposes. Researchers interested in using our data must provide a summary of the research they intend to conduct. The reviews will be completed within 2 weeks and then a decision will be sent to the applicant. The data are not publicly available due to hospital regulation restrictions.

## Code availability

The code and example data used in this study can be accessed at GitHub (https://github.com/jiangjiewei/Keratitis-Source).
