# Supplementary material for: Preventing corneal blindness caused by keratitis using artificial intelligence
Source: Nat Commun. 2021 Jun 18;12:3738. doi: 10.1038/s41467-021-24116-6 (PMC8213803; doi:10.1038/s41467-021-24116-6)
Supplement: Supplementary file 1 — Supplementary Information [file 41467_2021_24116_MOESM1_ESM.pdf]

Title: Preventing corneal blindness caused by keratitis using artificial intelligence

Supplementary Information

1. **Supplementary Figure 1. Visualization by t-distributed stochastic neighbor embedding (t-SNE) of the separability for the features learned by deep learning algorithms in the external test datasets. a** Zhejiang Eye Hospital (ZEH) dataset. **b** Jiangdong Eye Hospital (JEH) dataset. **c** Ningbo Ophthalmic Center (NOC) dataset. **d** "Smartphone" indicates the smartphone dataset. "Normal" indicates normal cornea. "Others" indicates cornea with other abnormalities.

2. **Supplementary Figure 2. Confusion matrices of three deep learning algorithms in external test datasets. a** Zhejiang Eye Hospital (ZEH) dataset. **b** Jiangdong Eye Hospital (JEH) dataset. **c** Ningbo Ophthalmic Center (NOC) dataset. **d** "Smartphone" indicates the smartphone dataset. "Normal" indicates normal cornea. "Others" indicates cornea with other abnormalities.

3. **Supplementary Figure 3. Classification errors by the deep learning system in both internal and external test datasets. a** Misclassification of "keratitis" by the deep learning system. **b** Misclassification of "cornea with other abnormalities" by the deep learning system. **c** Misclassification of "normal cornea" by the deep learning system.

4. **Supplementary Figure 4. Performance of the best deep learning algorithm DenseNet121 in the external datasets with and without poor-quality images. a** Zhejiang Eye Hospital (ZEH) dataset. **b** Jiangdong Eye Hospital (JEH) dataset. **c** Ningbo Ophthalmic Center (NOC) dataset. **d** "Smartphone" indicates the smartphone dataset. "Normal" indicates normal cornea. "Others" indicates cornea with other abnormalities. DEPI, datasets excluding poor-quality images. DIPI, datasets including poor-quality images.

5. **Supplementary Figure 5. Relationship between the misclassification rates and predicted probability values.** Normal" indicates normal cornea. "Others" indicates cornea with other abnormalities. The misclassification rate is the fraction of misclassified images in each predicted probability interval between the breaking points.

6. **Supplementary Table 1. Performance of the best deep learning system vs. corneal specialists in the ZEH dataset.**

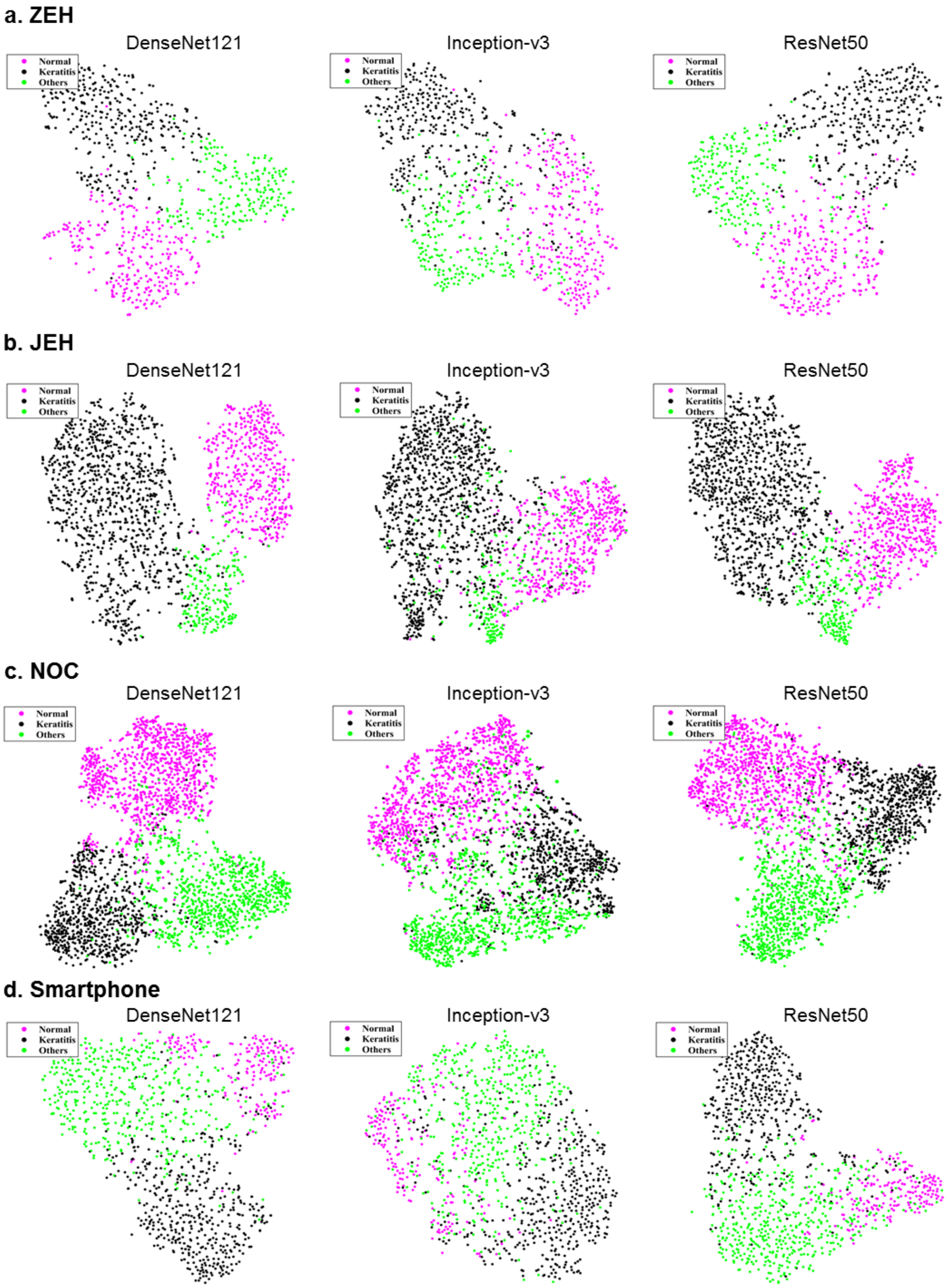

7. **Supplementary Figure 1. Visualization by t-distributed stochastic neighbor embedding (t-SNE) of the separability for the features learned by deep learning algorithms in the external test datasets. a** Zhejiang Eye Hospital (ZEH) dataset. **b** Jiangdong Eye Hospital (JEH) dataset. **c** Ningbo Ophthalmic Center (NOC) dataset. **d** "Smartphone" indicates the smartphone dataset. "Normal" indicates normal cornea. "Others" indicates cornea with other abnormalities.

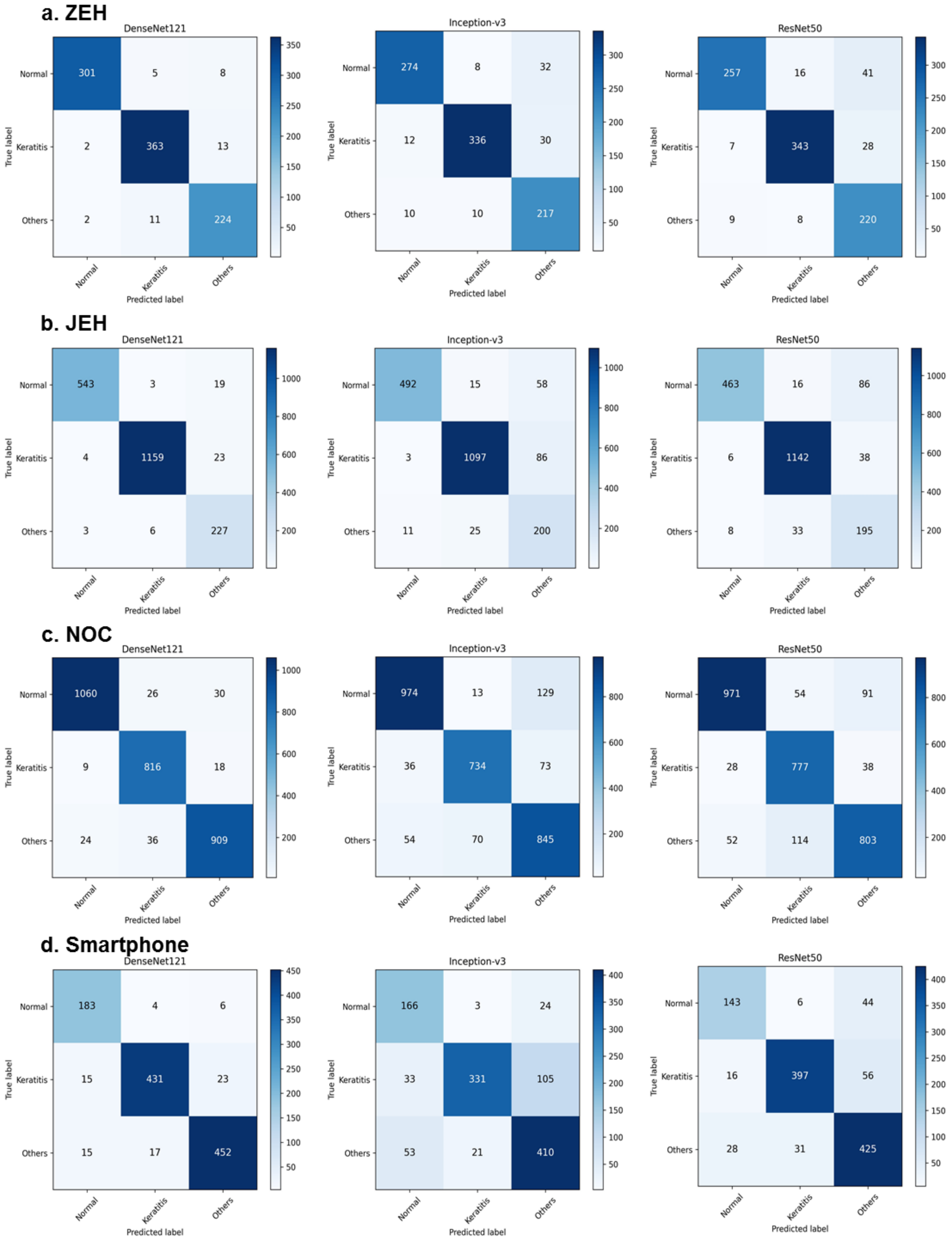

8. **Supplementary Figure 2. Confusion matrices of three deep learning algorithms in external test datasets. a** Zhejiang Eye Hospital (ZEH) dataset. **b** Jiangdong Eye Hospital (JEH) dataset. **c** Ningbo Ophthalmic Center (NOC) dataset. **d** “Smartphone” indicates the smartphone dataset. “Normal” indicates normal cornea. “Others” indicates cornea with other abnormalities.

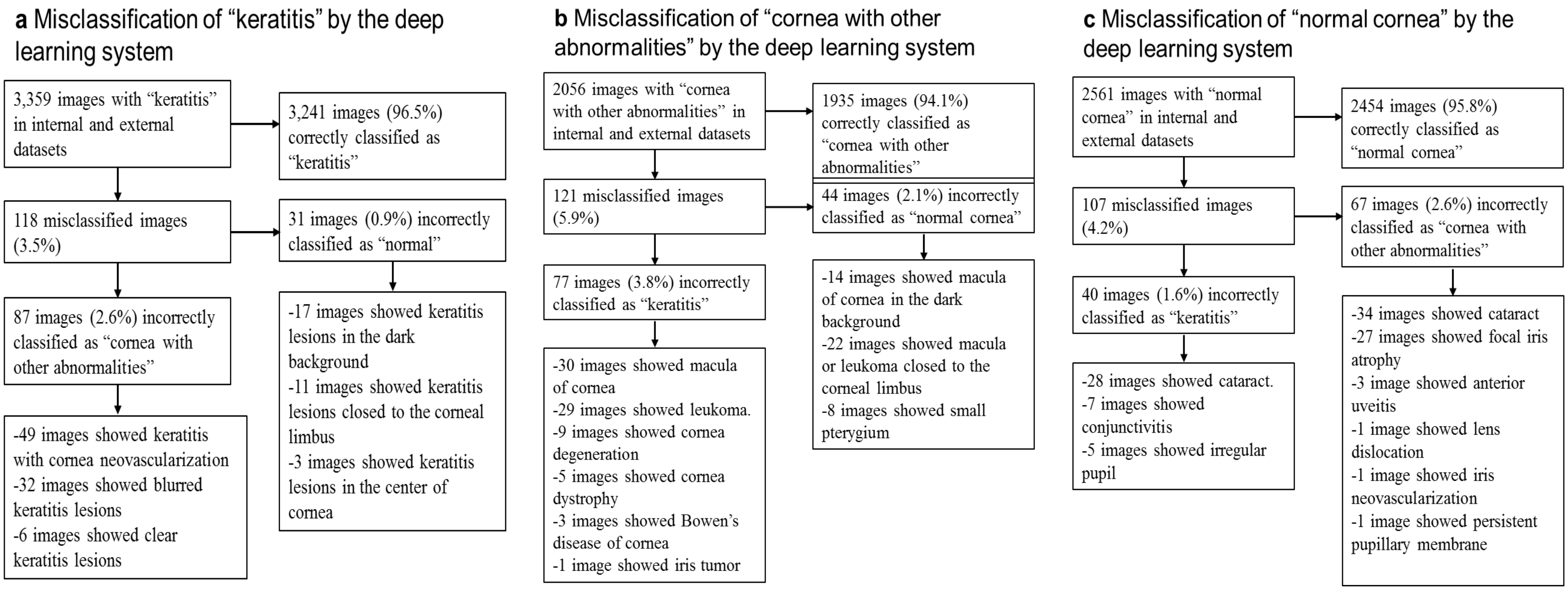

Supplementary Figure 3. Classification errors by the deep learning system in both internal and external test datasets. a Misclassification of "keratitis" by the deep learning system. **b** Misclassification of "cornea with other abnormalities" by the deep learning system. **c** Misclassification of "normal cornea" by the deep learning system.

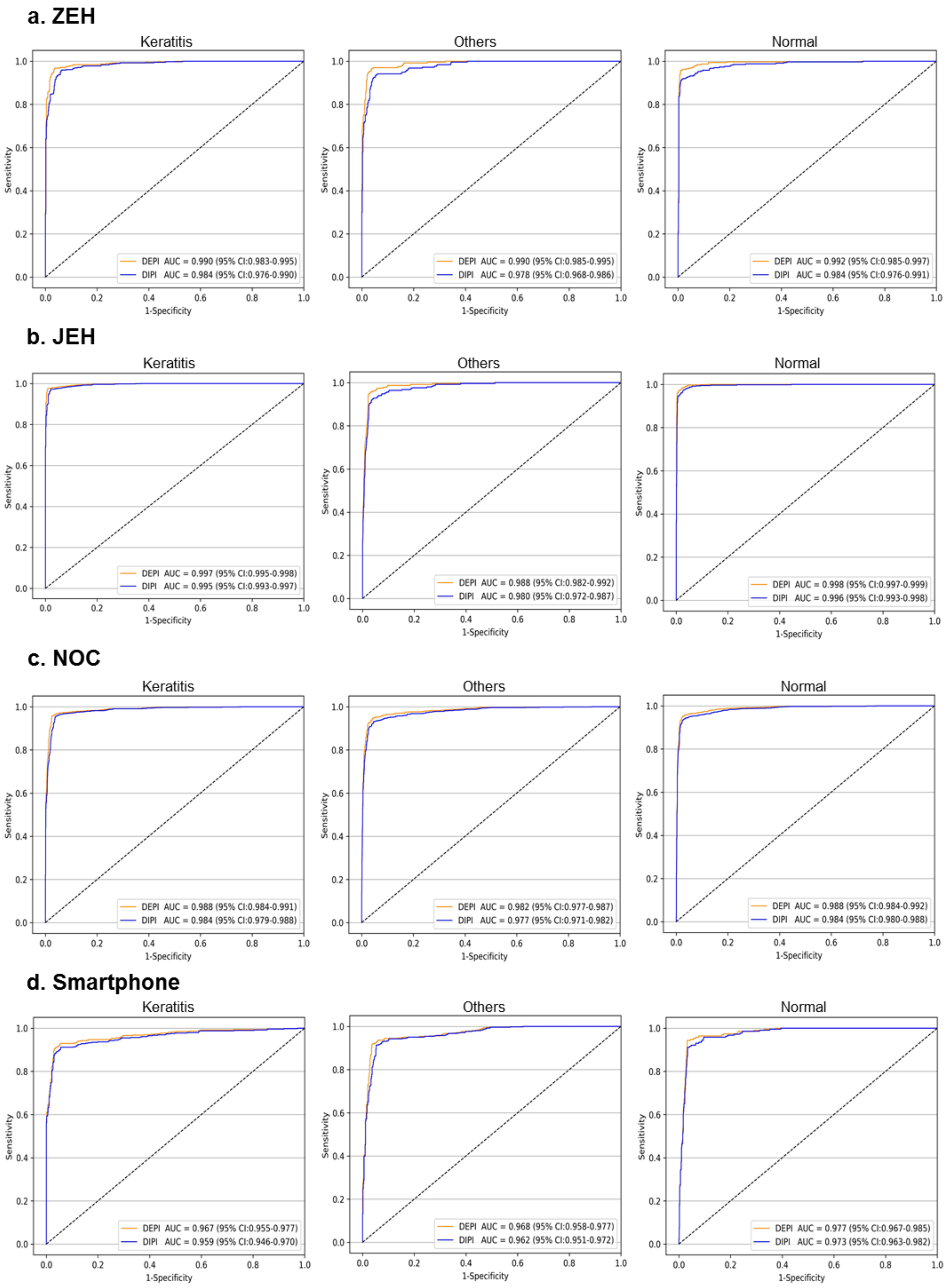

a. ZEH
Keratitis
DEPI AUC = 0.990 (95% CI:0.983-0.995)
DIPI AUC = 0.984 (95% CI:0.976-0.990)
Others
DEPI AUC = 0.990 (95% CI:0.985-0.995)
DIPI AUC = 0.978 (95% CI:0.968-0.986)
Normal
DEPI AUC = 0.992 (95% CI:0.985-0.997)
DIPI AUC = 0.984 (95% CI:0.976-0.991)
b. JEH
Keratitis
DEPI AUC = 0.997 (95% CI:0.995-0.998)
DIPI AUC = 0.995 (95% CI:0.993-0.997)
Others
DEPI AUC = 0.988 (95% CI:0.982-0.992)
DIPI AUC = 0.980 (95% CI:0.972-0.987)
Normal
DEPI AUC = 0.998 (95% CI:0.997-0.999)
DIPI AUC = 0.996 (95% CI:0.993-0.998)
c. NOC
Keratitis
DEPI AUC = 0.988 (95% CI:0.984-0.991)
DIPI AUC = 0.984 (95% CI:0.979-0.988)
Others
DEPI AUC = 0.982 (95% CI:0.977-0.987)
DIPI AUC = 0.977 (95% CI:0.971-0.982)
Normal
DEPI AUC = 0.988 (95% CI:0.984-0.992)
DIPI AUC = 0.984 (95% CI:0.980-0.988)
d. Smartphone
Keratitis
DEPI AUC = 0.967 (95% CI:0.955-0.977)
DIPI AUC = 0.959 (95% CI:0.946-0.970)
Others
DEPI AUC = 0.968 (95% CI:0.958-0.977)
DIPI AUC = 0.962 (95% CI:0.951-0.972)
Normal
DEPI AUC = 0.977 (95% CI:0.967-0.985)
DIPI AUC = 0.973 (95% CI:0.963-0.982)
Sensitivity
1-Specificity

Supplementary Figure 4. Performance of the best deep learning algorithm DenseNet121 in the external datasets with and without poor-quality images. a Zhejiang Eye Hospital (ZEH) dataset. **b** Jiangdong Eye Hospital (JEH) dataset. **c** Ningbo Ophthalmic Center (NOC) dataset. **d** "Smartphone" indicates the smartphone dataset. "Normal" indicates normal cornea. "Others" indicates cornea with other abnormalities. DEPI, datasets excluding poor-quality images. DIPI, datasets including poor-quality images.

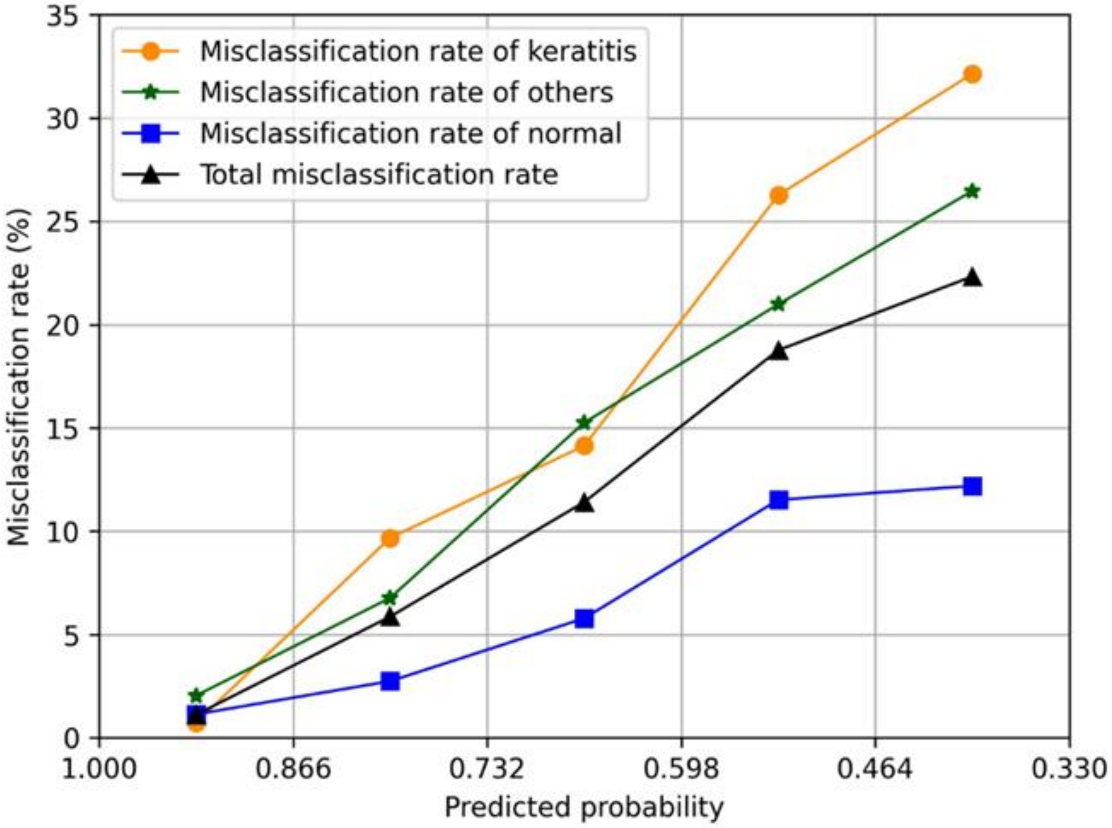

Supplementary Figure 5. Relationship between the misclassification rates and predicted probability values. Normal" indicates normal cornea. "Others" indicates cornea with other abnormalities. The misclassification rate is the fraction of misclassified images in each predicted probability interval between the breaking points.

Supplementary Table 1. Performance of the best deep learning system vs. corneal specialists in the ZEH dataset.

	Deep learning system	Corneal specialist A	Corneal specialist B	P1	P2
**Keratitis vs. others + normal**					
Sensitivity (95% CI)	96.0% (94.1-98.0)	95.0% (92.8-97.2)	97.1% (95.4-98.8)	.600	.549
Specificity (95% CI)	97.1% (95.7-98.5)	97.1% (95.7-98.5)	97.5% (96.1-98.8)	1.000	.853
Accuracy (95% CI)	96.7% (95.5-97.8)	96.2% (95.0-97.5)	97.3% (96.3-98.3)	.707	.497
**Others vs. keratitis + normal**					
Sensitivity (95% CI)	94.5% (91.6-97.4)	92.4% (89.0-95.8)	93.7% (90.6-96.8)	.457	.846
Specificity (95% CI)	97.0% (95.7-98.2)	96.1% (94.7-97.5)	97.5% (96.4-98.7)	.463	.622
Accuracy (95% CI)	96.3% (95.1-97.5)	95.2% (93.8-96.5)	96.6% (95.4-97.7)	.250	.900
**Normal vs. keratitis + others**					
Sensitivity (95% CI)	95.9% (93.7-98.1)	96.5% (94.5-98.5)	97.1% (95.3-99.0)	.835	.515
Specificity (95% CI)	99.3% (98.7-100)	99.2% (98.5-99.9)	99.3% (98.7-100)	1.000	1.000
Accuracy (95% CI)	98.2% (97.3-99.0)	98.3% (97.4-99.1)	98.6% (97.8-99.4)	1.000	.581

P1 refers to the *p*-value that was calculated between the deep learning system and corneal specialist A using the two-sided McNemar test. P2 refers to the *p*-value that was calculated between the deep learning system and corneal specialist B using the two-sided McNemar test. ZEH, Zhejiang Eye Hospital. Cornea specialist A has 3 years of clinical experience. Cornea specialist B has 6 years of clinical experience.
