# Supplementary material for: Preventing corneal blindness caused by keratitis using artificial intelligence
Source: Nat Commun. 2021 Jun 18;12:3738. doi: 10.1038/s41467-021-24116-6 (PMC8213803; doi:10.1038/s41467-021-24116-6)
Supplement: Supplementary file 2 — Reporting Summary [file 41467_2021_24116_MOESM2_ESM.pdf]

nature research

Corresponding author(s): Wei Chen

Last updated by author(s): May 28, 2021

Reporting Summary

Nature Research wishes to improve the reproducibility of the work that we publish. This form provides structure for consistency and transparency in reporting. For further information on Nature Research policies, see our Editorial Policies and the Editorial Policy Checklist.

Statistics

For all statistical analyses, confirm that the following items are present in the figure legend, table legend, main text, or Methods section.

n/a	Confirmed	
☐	☒	The exact sample size (*n*) for each experimental group/condition, given as a discrete number and unit of measurement
☐	☒	A statement on whether measurements were taken from distinct samples or whether the same sample was measured repeatedly
☐	☒	The statistical test(s) used AND whether they are one- or two-sided<br>*Only common tests should be described solely by name; describe more complex techniques in the Methods section.*
☒	☐	A description of all covariates tested
☒	☐	A description of any assumptions or corrections, such as tests of normality and adjustment for multiple comparisons
☐	☒	A full description of the statistical parameters including central tendency (e.g. means) or other basic estimates (e.g. regression coefficient) AND variation (e.g. standard deviation) or associated estimates of uncertainty (e.g. confidence intervals)
☐	☒	For null hypothesis testing, the test statistic (e.g. *F*, *t*, *r*) with confidence intervals, effect sizes, degrees of freedom and *P* value noted<br>*Give P values as exact values whenever suitable.*
☒	☐	For Bayesian analysis, information on the choice of priors and Markov chain Monte Carlo settings
☒	☐	For hierarchical and complex designs, identification of the appropriate level for tests and full reporting of outcomes
☐	☒	Estimates of effect sizes (e.g. Cohen's *d*, Pearson's *r*), indicating how they were calculated

Our web collection on statistics for biologists contains articles on many of the points above.

Software and code

Policy information about availability of computer code

Data collection	No software was used for data collection.
Data analysis	Three state-of-the-art convolutional neural network (CNN) architectures (DenseNet121, Inception-v3, and ResNet50) were investigated in this study. Deep learning models were trained using PyTorch (version 1.6.0) as a backend. The adaptive moment estimation (ADAM) optimizer with a 0.001 initial learning rate, β1 of 0.9, β2 of 0.999, and weight decay of 1e-4 was used. Statistical analyses were conducted using Python 3.7.8 (Wilmington, Delaware, USA). The 95% confidence intervals (CIs) for sensitivity, specificity, and accuracy were calculated with the Wilson Score approach using a Statsmodels package (version 0.11.1). ROC curves were created using the packages of Scikit-learn (version 0.23.2) and Matplotlib (version 3.3.1).<br>The code used in this study is available at https://github.com/jiangjiewei/Keratitis-Source.

For manuscripts utilizing custom algorithms or software that are central to the research but not yet described in published literature, software must be made available to editors and reviewers. We strongly encourage code deposition in a community repository (e.g. GitHub). See the Nature Research guidelines for submitting code & software for further information.

Data

Policy information about availability of data

All manuscripts must include a data availability statement. This statement should provide the following information, where applicable:

- Accession codes, unique identifiers, or web links for publicly available datasets
- A list of figures that have associated raw data
- A description of any restrictions on data availability

The code and example data used in this study is available at https://github.com/jiangjiewei/Keratitis-Source. The datasets generated and/or analyzed during the current study are available upon reasonable request from the corresponding author. Correspondence and requests for data materials should be addressed to WC

April 2020

(chenwei@eye.ac.cn). The datasets are not publicly available due to hospital regulation restrictions.

Field-specific reporting

Please select the one below that is the best fit for your research. If you are not sure, read the appropriate sections before making your selection.

- [x] Life sciences
- [ ] Behavioural & social sciences
- [ ] Ecological, evolutionary & environmental sciences

For a reference copy of the document with all sections, see nature.com/documents/nr-reporting-summary-flat.pdf

Life sciences study design

All studies must disclose on these points even when the disclosure is negative.

Sample size	For image classification using deep learning, a rule of thumb is 1,000 images per class, where this number can go down significantly if one uses pre-trained models. In this study, the parameters of each deep learning network were initialized using the ImageNet dataset pre-trained model. Besides, after removing 1,197 images without sufficient diagnostic certainty and 594 poor quality images, a total of 13,557 qualified images (6,055 images of keratitis, 2,777 images of the cornea with other abnormalities, and 4,725 images of the normal cornea) from 4 clinical centers (NEH, ZEH,JEH, and NOC) were used to develop and evaluate deep learning models. The sample size of our datasets is sufficient for the model training and testing.
Data exclusions	Images without sufficient evidence to determine a diagnosis and poor- quality images were excluded from the study.
Replication	Deep learning models were trained using PyTorch as a backend. The adaptive moment estimation (ADAM) optimizer with a 0.001 initial learning rate, β1 of 0.9, β2 of 0.999, and weight decay of 1e-4 was used. Each model was trained for 80 epochs. During the training process, validation loss was assessed on the validation dataset after each epoch and used as a reference for model selection. Each time the validation loss decreased, a checkpoint saved the model state and corresponding weight matrix. The model state with the lowest validation loss was saved as the final state of the model for use on the test dataset.
Randomization	The slit-lamp images drawn from the NEH dataset were randomly divided (7:1.5:1.5) into training, validation, and test datasets. Images from the same individual were assigned to only one same set for preventing leakage and biased assessment of performance.
Blinding	The investigators were blinded to the group allocation during data collection and/or analysis. Cornea specialists were blinded to the outcomes of the deep learning system.

Reporting for specific materials, systems and methods

We require information from authors about some types of materials, experimental systems and methods used in many studies. Here, indicate whether each material, system or method listed is relevant to your study. If you are not sure if a list item applies to your research, read the appropriate section before selecting a response.

Materials & experimental systems

n/a	Involved in the study	
x		Antibodies
x		Eukaryotic cell lines
x		Palaeontology and archaeology
x		Animals and other organisms
	x	Human research participants
x		Clinical data
x		Dual use research of concern

Methods

n/a	Involved in the study	
x		ChIP-seq
x		Flow cytometry
x		MRI-based neuroimaging

Human research participants

Policy information about studies involving human research participants

Population characteristics	The slit-lamp dataset included individuals who presented for ocular surface disease examination, ophthalmology consultations, and routine ophthalmic health evaluations. The smartphone dataset was derived from Wenzhou Eye Study which aimed to detect ocular surface diseases using smartphones.
Recruitment	A total of 7,120 slit-lamp images collected from NEH were used to develop a deep learning system. Four additional datasets including 6,925 slit-lamp images and 1,303 smartphone-based cornea images were utilized to externally test the system.
Ethics oversight	This retrospective study was approved by the Institutional Review Board of Ningbo Eye Hospital (NEH) and conducted in accordance with the tenets of the Declaration of Helsinki.

Note that full information on the approval of the study protocol must also be provided in the manuscript.
